# Differentiable Network Pruning via Polarization of Probabilistic Channelwise Soft Masks

**DOI:** 10.1155/2022/7775419

**Published:** 2022-05-05

**Authors:** Ming Ma, Jiapeng Wang, Zhenhua Yu

**Affiliations:** ^1^School of Information Engineering, Ningxia University, Yinchuan 750021, China; ^2^Collaborative Innovation Center for Ningxia Big Data and Artificial Intelligence Co-Founded By Ningxia Municipality and Ministry of Education, Yinchuan 750021, China

## Abstract

Channel pruning has been demonstrated as a highly effective approach to compress large convolutional neural networks. Existing differentiable channel pruning methods usually use deterministic soft masks to scale the channelwise outputs and explore an appropriate threshold on the masks to remove unimportant channels, which sometimes causes unexpected damage to the network accuracy when there are no sweet spots that clearly separate important channels from redundant ones. In this article, we introduce a new differentiable channel pruning method based on polarization of probabilistic channelwise soft masks (PPSMs). We use variational inference to approximate the posterior distributions of the masks and simultaneously exploit a polarization regularization to push the probabilistic masks towards either 0 or 1; thus, the channels with near-zero masks can be safely eliminated with little hurt on network accuracy. Our method significantly relieves the difficulty faced by the existing methods to find an appropriate threshold on the masks. The joint inference and polarization of probabilistic soft masks enable PPSM to yield better pruning results than the state of the arts. For instance, our method prunes 65.91% FLOPs of ResNet50 on the ImageNet dataset with only 0.7% model accuracy degradation.

## 1. Introduction

Convolutional neural network (CNN) yields unprecedented success in computer vision tasks [1, 2], due to its intrinsic ability of automatically learning meaningful features. To achieve better results on these tasks, the structure of CNN is expanding wider and deeper. However, the performance elevation is often accompanied with extensive consumption of memory and computation footprint, which inhibits the deployment of complex CNNs on resource-constrained devices. Network compression techniques [3–5] have relieved the issue through condensing a large CNN into a compact subnetwork (subnet), and channel pruning is deemed as one of the most effective methods for network compression.

Channel pruning aims at removing semantically redundant channels from a pretrained or a baseline network with little damage to the model accuracy. Early works on channel pruning mainly employ an iterative search-evaluation scheme comprising of generation of subnets and evaluation of the subnets on a validation set. For instance, He et al. [6] propose to sequentially prune each layer of the network based on LASSO regression, and AMC [7] generates the reserved channels in each layer via reinforcement learning. The layerwise pruning may result in suboptimal solution due to deficient representation of global structural information of the network. To improve subnet search accuracy and efficiency, Cao et al. [8] leverage Bayesian optimization to generate the priority ordering of subnets for evaluation. Some methods exploit channel importance scores to guide the selection of subnets. For instance, Liebenwein et al. [9] use an important sampling distribution to yield subnets by giving higher sampling probability to important filters. LeGR [10] generates subnets with different trade-offs between model accuracy and efficiency by inferring global ranking of the filters. Similarly, HRank [11] sorts filters by the rank of feature maps and removes low-ranked filters to yield a subnet. These methods all explicitly or implicitly depend on empirically defined metrics to assess the importance of filters and usually need to explore a threshold of the scores to remove unimportant filters or channels.

To learn a well-performing subnet under an end-to-end manner, differentiable pruning has become a popular approach for channel pruning. The basic concept is attaching learnable masks or gate functions behind channels to scale the original outputs and utilizing certain regularizations on the masks or gates to get an importance ordering of all channels. Lin et al. [12] use binary masks to remove redundant filters by setting corresponding masks as 0. Such hard pruning gives rise to the difficulty of mask optimization. To relieve the training complexity, many methods based on soft pruning have been proposed in recent years [13–15]. For instance, GAL [16] employs soft masks to remove structural redundancy with adversarial learning, GBN [17] estimates filter importance ranking through exploring the effects of setting channelwise gate to zero on the loss function, and DMCP [18] formulates channel pruning of each layer as a Markov process that defines the retaining probability of each channel. Kim et al. [19] also introduce the concept of gates for differentiable channel pruning. While these soft pruning methods perform acceptably well, they still need to explore an appropriate threshold on the masks or gates to define unimportant channels, which sometimes causes unexpected damage to the network accuracy when there are no sweet spots that clearly separate the channels into two parts. In addition, the learning of deterministic masks or gates may suffer from low stability and deficient convergence in large networks.

**Figure 1:**
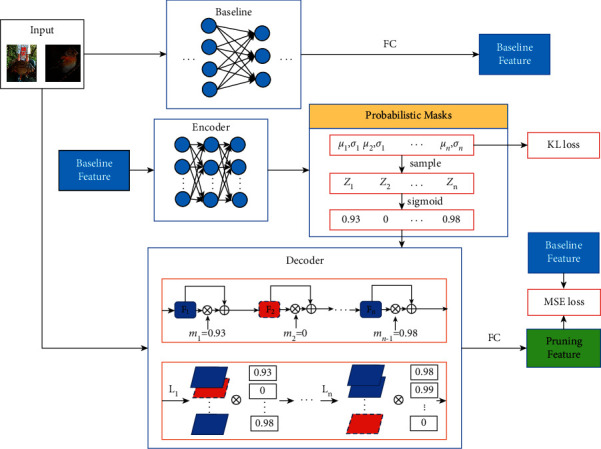
An illustration of PPSM. The PPSM framework for channel pruning consists of a conditional variational auto-encoder (CVAE), where the encoder learns the posterior distribution of channelwise soft masks given the output features of a baseline network, and the decoder formed by the pruned network learns to recover the baseline features. PPSM combines variational inference with a polarization regularization to effectively learn the posterior distributions of the masks and simultaneously divide the filters into two clearly separated parts, and therefore facilitate the pruning of channels with masks close to zero.

In this article, we introduce a new differentiable channel pruning method based on polarization of probabilistic soft masks (PPSMs).  Figure 1 provides an intuitive illustration of the idea of mask polarization. PPSM is built on the assumption that the global channel ranking may vary with the input (similar concept is adopted in dynamic pruning); therefore, deterministic masks used by existing methods cannot capture this input-aware property. We use probabilistic channelwise soft masks to implicitly represent the uncertainty on channel ranking. To enable stable learning of the masks, variational inference is exploited to approximate the posterior distributions of the masks given the output features of a baseline network. Meanwhile, a new polarization regularization is utilized to push masks of redundant and important channels towards 0 and 1, respectively; thus, the channels with near-zero masks can be safely eliminated with little hurt on network accuracy. Our method significantly relieves the difficulty faced by the existing methods to find an appropriate threshold on the masks. To the best of our knowledge, PPSM is the first method to make joint inference and polarization of probabilistic soft masks.

Our main contributions are summarized as follows:

1. We propose a differentiable channel pruning method to remove redundant channels from a baseline network through learning input-aware probabilistic channelwise soft masks.
2. Variational inference and polarization regularization are introduced to learn and push the probabilistic masks towards two ends and therefore clearly separate important channels from redundant ones.
3. Extensive evaluations of PPSM on popular network architectures and datasets show our method outperforms the state of the arts, and it prunes more FLOPs with less loss of model accuracy.

## 2. Related Works

### 2.1. Network Pruning

Network pruning eliminates the unnecessary weights or structured units such as filters and neurons of a pretrained neural network. Fine-grained pruning directly removes redundant weights within a filter or neuron and produces a highly sparse weight matrix. Many works [20, 21] mainly apply sparsity-induced penalty on the weights to remove insignificant weights. While nonstructured pruning greatly reduces the parameters of the network, it is not hardware-friendly and requires a specially designed sparsity matrix multiplication library for acceleration. By comparison, coarse-grained or structured pruning [22–25] aims at removing structured units such as filters, channels, or layers. The widely used strategy of structured pruning is to attach a learnable scaling factor or mask after each structure is pruned with sparsity regularization [26–28]. Jung et al. [29] propose a new real-time target tracking meta-learning framework with efficient model adaptation and channel pruning. He et al. [22] propose meta-attribute-based filter pruning (MFP), which adaptively selects the most appropriate pruning standard through an attribute (meta-attribute) of the current state of the neural network. Li et al. [23] propose a new fusion catalytic pruning method called FuPruner to simultaneously optimize parametric and nonparametric operators to accelerate neural networks. Some recent efforts use two or more different techniques for joint optimization. This provides another flexible option for network compression because the two technologies complement each other. The joint optimization of pruning and other model compression algorithms (such as quantization, knowledge distillation, and matrix decomposition) [30–32] can deal with a larger search space and obtain a more compact network. Recent works like joint-DetNAS [33] and NPAS [34] perform joint optimization of neural architecture search (NAS) and pruning.

### 2.2. Neural Architecture Search

NAS aims at automatically finding a compact neural architecture from a large search space. Early works use either reinforcement learning [35] or genetic algorithm [36] to update model responses for generating architectures with better performance. However, the search space of these methods is very large and significant computational overhead is required to search and select the best model from thousands of models. To address this problem, Differentiable Architecture Search (DARTS) [37] continuously processes the search space, which facilitates optimization algorithms such as gradient descent to find the optimal network structure. Our method can also be seen as a NAS process. Compared with conventional NAS, our method obtains the posterior distribution of the mask given the baseline output features and uses variational inference to learn the soft mask. Then, the polarization regularization of the soft mask is employed to remove the channels with soft masks close to zero, resulting in a compact network.

## 3. Method

### 3.1. Notations and Preliminaries

Given a batch of input images {**x**_*i*_}_*i*=1_^*N*^, the baseline network outputs corresponding feature maps {**y**_*i*_}_*i*=1_^*N*^ from the last layer. The input and output pairs {(**x**_*i*_, **y**_*i*_)}_*i*=1_^*N*^ constitute a training dataset for supervised channel pruning. We use **F**_*i*_ ∈ *ℝ*^*C*_*i*_×*W*_*i*_×*H*_*i*_^ to denote the feature map derived from the *i*-th layer, where *i* is the layer index, *C*_*i*_ is the number of channels, and *W*_*i*_ and *H*_*i*_ are the height and width of the feature map, respectively. Suppose the number of filters across the network is *n*, we use a *n*-dimensional variable **m**=(*m*^(1)^,…, *m*^(*n*)^) to represent the soft masks, where each element *m*^(*i*)^ ∈ [0,1]. By multiplying the channelwise outputs of the baseline network by the soft masks, we can get a pruned network through setting certain masks to 0. For each input **x**_*i*_, the corresponding soft masks are denoted as **m**_*i*_, and the output feature map of the pruned network is optimized to approximate the baseline **y**_*i*_.

### 3.2. Probabilistic Soft Masks

The usage of probabilistic masks is motivated by the instance-aware channel ranking used in dynamic pruning. A single deterministic mask cannot capture such dynamics, while a distribution is more effective to characterize the variance of channel importance in static network pruning. In addition, learning a distribution tends to have better stability than learning a single deterministic value. Therefore, probabilistic soft mask is used to capture the variance of channel importance. Given an input sample **x**_*i*_, we assume the output **y**_*i*_ can be well approximated by cancelling out certain filters. Based on these conceptions, we formulate the dependence of output feature map **y**_*i*_ on input **x**_*i*_ and soft masks **m**_*i*_ with a deep conditional generative model (CGM): for given input **x**_*i*_, sampled **m**_*i*_ from the prior distribution *p*_*θ*_(**m**_*i*_*| ***x**_*i*_) and generated output **y**_*i*_ from the distribution *p*_*θ*_(**y**_*i*_*| ***x**_*i*_, **m**_*i*_). Direct training of the deep CGM to maximize the conditional log-likelihood is intractable; therefore, we employ stochastic gradient variational Bayes (SGVB) [38] to optimize the variational lower bound on the conditional log-likelihood:

(1) $$\begin{array}{c} \log\;p_{\theta }\left(y_{i}|x_{i}\right)\geq -\text{KL}\left(q_{\phi }{\left(m_{i}|x_{i},y_{i}\right)\|}_{\theta }\left(m_{i}|x_{i}\right)\right)\\ +E_{q_{\phi }\left(m_{i}|x_{i},y_{i}\right)}\left[\log\;p_{\theta }\left(y_{i}|x_{i},m_{i}\right)\right], \end{array}$$

where *ϕ* and *θ* are variational and generative parameters, respectively. The KL divergence measures the similarity between the approximate and true posteriors.

To simplify the computation, we further assume the posterior distribution of **m**_*i*_ is only conditioned on **y**_*i*_, that is, *q*_*ϕ*_(**m**_*i*_*| ***x**_*i*_, **y**_*i*_)=*q*_*ϕ*_(**m**_*i*_*| ***y**_*i*_). We adopt a simplified form of the conditional probability based on two reasons: (1) although the baseline network may give same outputs for different inputs, this will rarely happen given the complex nonlinear property of the network; (2) even if the output features of two images are same, the images are most likely from the same class and have little semantic difference. Therefore, we remove **x**_*i*_ from conditional probability *q*_*ϕ*_(**m**_*i*_*| ***x**_*i*_, **y**_*i*_) to simplify the model training. As the element value of **m**_*i*_ is constrained to the interval [0, 1], directly approximating the posterior distribution of **m**_*i*_ is computationally inconvenient; therefore, we introduce an auxiliary *n*-dimensional real-valued variable **z**_*i*_, to calculate **m**_*i*_ by applying sigmoid function to each element of **z**_*i*_, which we denote as **m**_*i*_=*S*(**z**_*i*_). Based on these definitions, the lower bound can now be formulated as

(2) $$\begin{array}{c} ℒ\left(x_{i},y_{i};\phi ,\theta \right)=-\text{KL}\left(q_{\phi }\left(z_{i}|y_{i}\right)\|p_{\theta }\left(z_{i}|x_{i}\right)\right)+E_{q_{\phi }\left(z_{i}|y_{i}\right)}\left[\log\;p_{\theta }\left(y_{i}|x_{i},z_{i}\right)\right]. \end{array}$$

We then leverage conditional variational auto-encoder (CVAE) to optimize the lower bound with respect to both *ϕ* and *θ*.  Figure 1 shows the proposed PPSM framework that is built on the CVAE to reason the probabilistic soft masks. The encoder consists of 5 fully connected layers of which the last two layers output the mean and variance of each **z**_*i*_, and the decoder is the pruned network that has same structure to the baseline network. Specifically, we use a centered isotropic multivariate Gaussian for the conditional prior on **z**_*i*_ with *p*_*θ*_(**z**_*i*_*| ***x**_*i*_)=*𝒩*(**z**_*i*_; 0, **I**) and also assume the variational posterior is a multivariate normal distribution with diagonal covariance matrix: *q*_*ϕ*_(**z**_*i*_*|| ***y**_*i*_)=*𝒩*(**z**_*i*_; *μ*_*i*_, *σ*_*i*_^2^**I**), where *μ*_*i*_ and *σ*_*i*_ are the outputs of the encoder and represents the mean and s.d. of the posterior, respectively. We sample **z**_*i*_ from the posterior *q*_*ϕ*_(**z**_*i*_*| ***y**_*i*_) using **z**_*i*_=*g*_*ϕ*_(**y**_*i*_, *ϵ*)=*μ*_*i*_+*σ*_*i*_⊙*ϵ*, *ϵ* ~ *𝒩*(0, **I**), where ⊙ denotes element-wise product. The soft masks **m**_*i*_ are then calculated using **m**_*i*_=*S*(**z**_*i*_).

We use the mean soft masks **m**=1/*N*∑_*i*_**m**_*i*_ to scale the channelwise feature maps for each input **x**_*i*_. Here, **m** represents an average contribution of the inputs to channel importance and shows less variance than **m**_*i*_,  1 ≤ *i* ≤ *N*, and therefore is easier to be optimized. Given the soft masks **m** and input **x**_*i*_, the decoder yields the reconstructed feature map *f*(**x**_*i*_, **m**; *θ*) to approximate the baseline **y**_*i*_. Specifically, we use MSE loss to align the outputs of the pruned and baseline networks:

(3) $$\begin{array}{c} {ℒ}_{\text{MSE}}=\frac{1}{2N}\sum_{i=1}^{N}{\left\|f\left(x_{i},m;\theta \right)-y_{i}\right\|}_{2}^{2}. \end{array}$$

The optimization objective now becomes minimizing the following loss function:

(4) $$\begin{array}{c} {ℒ}_{\text{CVAE}}=\sum_{i=1}^{N}\text{KL}\left(q_{\phi }\left(z_{i}|y_{i}\right)\|p_{\theta }\left(z_{i}|x_{i}\right)\right)+{ℒ}_{\text{MSE}}. \end{array}$$

The CVAE loss function makes it convenient to differentially approximate the posteriors of the soft masks and effectively recovering the baseline features.

### 3.3. Polarization Regularization

Optimization of ℒ_CVAE_ does not provide a guarantee of clear separation of important filters from redundant ones; therefore, appropriate regularization on the soft masks is essential for harmless channel pruning. The conventional strategies that use either L1 or L2 regularization [39] aim to minify the masks of unimportant filters and need to carefully explore a threshold on the masks to prune filters with masks below the threshold. Inspired by the work in [28], we introduce a polarization regularizer on the probabilistic soft masks to push the posteriors of the masks towards 0 or 1, such that sweet spots that clearly separate the channels into two parts can be easily found. The adopted polarization regularizer is defined as follows:

(5) $$\begin{array}{c} R\left(m\right)=t{\left\|m\right\|}_{1}-{\left\|m-\bar{m}1_{n}\right\|}_{1}, \end{array}$$

where $\bar{m}$ denotes the mean of *m*^(1)^,…, *m*^(*n*)^. The effect of the second RHS term $-{\left\|m-\bar{m}1_{n}\right\|}_{1}$ is to keep *m*^(*i*)^,  1 ≤ *i* ≤ *n* as far away from the mean as possible. The term $-{\left\|m-\bar{m}1_{n}\right\|}_{1}$ gets its extremums at vertices of the *n*-dimensional cube [0,1]^*n*^, and the minimum is reached if half elements of **m** are 0. The hyperparameter *t* is introduced to control the weight of L1 regularization and also determine the sparsity of the soft masks.

### 3.4. Optimization

By combining the loss functions associated with the CVAE, polarization regularizer, and regularizations on parameters *ϕ* and *θ*, we derive the following objective function:

(6) $$\begin{array}{c} \min_{\phi ,\theta }{ℒ}_{\text{CVAE}}+\lambda R\left(m\right)+\lambda_{\phi }R\left(\phi \right)+\lambda_{\theta }R\left(\theta \right), \end{array}$$

where *R*(*ϕ*) is L2 regularization on variational parameters *ϕ*, *R*(*θ*) is L2 regularization on generative parameters *θ*, and the weights *λ*_*ϕ*_ and *λ*_*θ*_ are fixed to 5*e* − 4. We can optimize the objective function with respect to *ϕ* and *θ* using a differentiable algorithm such as stochastic gradient descent (SGD).

### 3.5. Pruning Strategy

After the model converges, the distribution of soft masks is analyzed to identify unimportant filters. Given a batch of input images, we measure the expected value of the mean soft masks **m**:

(7) $$\begin{array}{c} \bar{m}=E_{q_{\phi }\left(z_{i}|y_{i}\right)}\left[m\right]=\frac{1}{N}\sum_{i=1}^{N}E_{q_{\phi }\left(z_{i}|y_{i}\right)}\left[m_{i}\right],=\frac{1}{N}\sum_{i=1}^{N}\int q_{\phi }\left(z_{i}|y_{i}\right)S\left(z_{i}\right)\text{d}z_{i}. \end{array}$$

**Figure 2:**
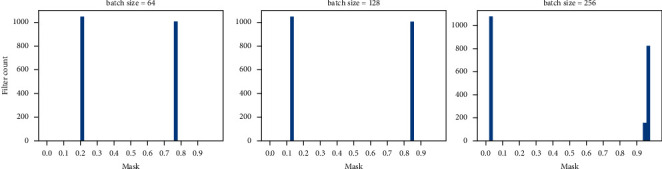
Analysis of the effect of batch size on learning the probabilistic masks. Batch sizes of 64, 128, and 256 were explored. The results show the soft masks were clearly separated into two parts across different batch sizes.

**Figure 3:**
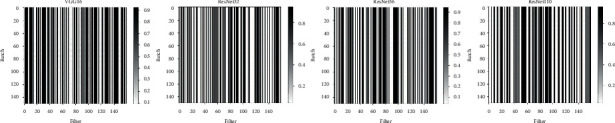
Investigation of the consistency of learned soft masks across different input batches. For a given filter, values of the mask learned from different batches are compared. The experiments were conducted on VGG16, ResNet32, ResNet56, and ResNet110 using the CIFAR-10 dataset. The soft masks of randomly sampled 150 filters are analyzed across 150 batches.

As *q*_*ϕ*_(**z**_*i*_*| ***y**_*i*_)*S*(**z**_*i*_) forms a complex function with respect to **z**_*i*_, the integral is intractable to calculate; therefore, we get Monte Carlo estimate of the expectation of **m**_*i*_ as follows:

(8) $$\begin{array}{c} E_{q_{\phi }\left(z_{i}|y_{i}\right)}\left[m_{i}\right]\;≃\;\frac{1}{L}\sum_{l=1}^{L}S\left(g_{\phi }\left(y_{i},{ɛ}_{l}\right)\right),\;{ɛ}_{l}\in N\left(0,I\right), \end{array}$$

where *L* is the number of samples. Each element of $\bar{m}$ denotes the soft mask attached to one of the filters. By utilizing the polarization effect, we do not need to explore a threshold on soft masks and can directly set the threshold as 0.5 to prune filters. When investigating the distribution histogram of $\bar{m}$, a bimodal distribution is always observed and two peaks are clearly separated: one locates close to 0, and the other locates close to 1 (illustrated in Figure 2). In addition, the filters are completely separated into two parts with a large margin. We also observe that the batch of inputs has little effect on the distribution of soft masks after the model converges (demonstrated in Figure 3); therefore, only one batch of inputs is required when pruning the filters.

## 4. Experiments

### 4.1. Experimental Settings

We evaluated the proposed method on two datasets: CIFAR-10 [40] and ImageNet ILSVRC 2012 [41]. CIFAR-10 is a 10-class image classification dataset with an image size of 32 × 32. It contains 50k training images and 10k validation images. ImageNet is a large-scale image classification dataset, which contains 1.28 million training images and 50k validation images. On CIFAR-10, we evaluated the proposed method on VGG-16 [42] and ResNets [43] (including ResNet32, ResNet56, and ResNet110). On ImageNet dataset, we assessed our method on ResNet50 and MobileNet v2 [44].

#### 4.1.1. Implementation Details

All networks were trained from scratch. The same data augmentation strategies were used as done in PyTorch official examples [45]. The training was conducted to run 200 and 100 epochs on CIFAR-10 and ImageNet datasets, respectively, with an initial learning rate of 0.1 and a mini-batch size of 128. The learning rate was multiplied by 0.1 at 50% and 75% of the training epochs on CIFAR-10, and multiplied by 0.1 at 30, 60, and 90 epochs on ImageNet. We utilized an SGD optimizer with a weight decay of 0.0005 and a momentum of 0.9. For MobileNet v2 on ImageNet, we used cosine annealing to automatically reduce the learning rate. All experiments were implemented on two NVIDIA RTX 3090 GPUs and Intel(R) Xeon(R) Gold 5218 CPU by PyTorch.

#### 4.1.2. Hyperparameter

During the pruning process, we need to set two hyperparameters *λ* and *t* to achieve desired FLOPs reduction. The hyperparameter *λ* controls the weight of polarization regularizer. With a larger *λ*, the soft mask will move more obviously to 0 and 1. The hyperparameter *t* controls the ratio of FLOPs to be reduced. A larger *t* will result in more FLOP reduction. In our experiments, we empirically set *λ*  = 0.0004 on CIFAR-10 and *λ*  = 0.00005 on ImageNet. To obtain the desired FLOPs reduction, different *t* values need to be tested for different network architectures (as shown in Table 1), and we set the range of *t* to [−2, 2]. For example, when pruning ResNet56 on CIFAR-10, we obtained FLOPs reduction by 54.6% at *t* = 0.2. In addition, the initial learning rate during fine-tuning was set to 0.01.

### 4.2. Results

#### 4.2.1. Results on CIFAR-10

We first compared our method to the state of the arts on a small-scale CIFAR-10 dataset. Channel pruning was performed on four popular neural networks including VGG16, ResNet32, ResNet56, and ResNet110, and the results are shown in Table 2.

**Figure 4:**
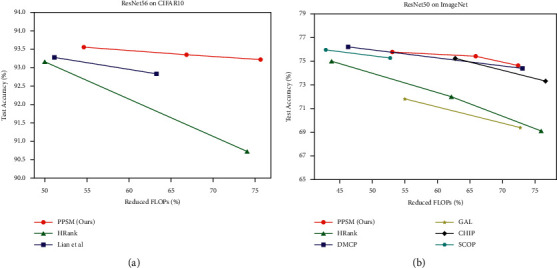
The change in the test accuracy of different methods with respect to the percent of reduced FLOPs. (a) shows the comparison results of pruning ResNet56 on CIFAR-10, and the pruning rate changes from 50% to 70%. (b) depicts the comparison results of pruning ResNet50 on ImageNet, and the pruning rate changes from 40% to 75%. PPSM is compared to the most representative existing methods.

When pruning VGG16, PPSM elevates the accuracy by 0.06% with 66.20% FLOPs pruned and performs better than HRank [11] and SCP [14] by yielding similar FLOP reduction. For ResNet32, when compared to LFPC [46] and Wang et al. [47], our method achieves the best accuracy at similar pruning rates of ∼53%, with an increase of 0.12% over baseline accuracy. In addition, PPSM outperforms LRF [27] and MainDP [15] by pruning 64.35% FLOPs and improves model accuracy by 0.09%. For ResNet56, PPSM was compared to 9 state-of-the-art methods in terms of high pruning rate (∼75% drop in FLOPs) and low pruning rate (∼50% drop in FLOPs), and our method performs better than or comparably to the competitors. For instance, with more FLOPs removed (75.62% vs 73.90%), PPSM exhibits lower accuracy loss (0.22% vs 0.26%) than LRF. Our method also increases the accuracy by 0.13% with 54.6% FLOPs compression, which is better than the results of DPFPS [49] and Wang et al. [47].  Figure 4(a) depicts the change in the test accuracy of different methods with respect to the percent of reduced FLOPs, and the results suggest PPSM achieves a higher accuracy than the competitors across different FLOP reduction rates. For ResNet110, with a similar FLOP reduction rate of ∼68.5%, our method performs much better than HRank in preserving network accuracy (−0.23% vs 0.85% accuracy loss). In addition, LRF improves model accuracy by 0.58% at 62.6% FLOP reduction, and PPSM prunes more FLOPs (68.7%) with 0.23% increase in model accuracy.

We utilize one batch of inputs to reason the distribution of the soft masks after the model converges and use the inferred distribution to determine the filters to prune. To investigate the effect of different batches of inputs on the distribution of the soft masks, we compared the inferred $\bar{m}$ of first 100 filters across 100 batches on VGG16, ResNet32, ResNet56, and ResNet110. The results in Figure 3 imply our method is robust to the change in batches and outputs highly consistent soft mask for each filter across different batches, suggesting the CVAE framework and polarization regularizer adopted in PPSM are beneficial to stabilizing the learning of probabilistic masks, therefore making PPSM well adaptive to different network architectures.

#### 4.2.2. Results on ImageNet

We further evaluated the performance of PPSM on large-scale ImageNet dataset, and also made comparisons to the state-of-the-art methods.

We evaluated the top-1 and top-5 accuracy and FLOP reduction rate of PPSM on ResNet50 and MobileNet V2 networks, and the results are shown in Table 3. To better verify the effectiveness of our method, the competitive methods we choose are from recently published works, such as GAL [16], HRank [11], Zhuang et al. [28], GBN [17], DMC [13], DMCP [18], SCP [14], SCOP [50], LRF [27], DPFPS [49], CHIP [51], and SRR-GR [48]. For ResNet50, we conducted experiments at pruning rates of 50%, 60%, and 70%. The results show PPSM surpasses other methods in top-1 and top-5 accuracy when FLOPs are reduced by 60% and 70%. Specifically, compared with GAL, HRank, and CHIP, PPSM has the maximum reduction rate of 65.91% in FLOPs, while its top-1 accuracy only decreases by 0.7% and top-5 accuracy only decreases by 0.32, which is significantly better than the results of other three methods. Similarly, when FLOPs are reduced by ∼70%, our method delivers higher top-1 and top-5 accuracies than other methods. With ∼55% FLOP reduction, LRF better recovers model accuracy than DMC, SCP, and SRR-GR. The pruning rate of LRF is slightly higher than that of PPSM (56.40% vs 53.07%), but the top-1 accuracy of PPSM decreases less than that of LRF (0.35% vs 0.50%). As shown in Figure 4(b), PPSM's accuracy is less sensitive to the FLOP reduction rate, whereas the accuracy of the existing most advanced methods decreases significantly as the pruning rate increases. For the lightweight network MobileNet v2, PPSM has the lowest decrease in accuracy after pruning. Our method removes ∼28% FLOPs with only 0.45% accuracy loss, while Metapruning [52] causes 0.80% drop in accuracy when 27% FLOPs are pruned, and DPFPS prunes ∼25% FLOPs with a cost of 0.9% accuracy loss.

Taken together, the superior performance of PPSM is attributed to the effective polarization of probabilistic soft masks in a CVAE framework, where the uncertainty on channel importance is well characterized by approximating posterior distribution of the soft masks.

### 4.3. Ablation Study

#### 4.3.1. The Effectiveness of the Probabilistic Mask

To verify the effectiveness of our adopted probabilistic masks, we compared the performance under two scenarios where probabilistic and deterministic masks are employed, respectively. For generating deterministic masks, we directly applied the sigmoid function on the *μ*_*i*_ outputted by the encoder of the CVAE and trained the model without KL loss.  Table 4 shows the comparison results of pruning ResNet32 and ResNet56 on the CIFAR-10 dataset. The results indicate probabilistic masks have advantages over deterministic masks in preserving the model capability across different pruning rates of FLOPs. For instance, the model accuracy is improved by 0.12% when reducing ∼53% FLOPs of ResNet32 with probabilistic masks, while decreased by 0.07% if deterministic masks are used. The superiority of probabilistic masks is more obvious when pruning ResNet56. The pruned network using probabilistic masks gains at least 0.28% accuracy improvement over the pruned model generated by deterministic masks. These results demonstrate probabilistic masks can effectively capture the uncertainty on channel importance and thus deliver more accurate identifications of important channels than the deterministic masks.

#### 4.3.2. The Effect of Batch Size on Learning the Probabilistic Masks

Polarization regularization encourages the masks to move towards both ends and results in a clear boundary between the two parts of separated filters and thus makes it easier to select a threshold to remove the less important filters. As PPSM gathers statistics of the soft masks from the images within a batch to prune the filters, we further examined the effect of batch size on channel pruning. Specifically, the results based on batch sizes of 64, 128, and 256 were compared. The results in Figure 2 suggest batch size has little effect on learning the distribution of the masks, and filters are clearly divided into two parts with soft masks close to either 0 or 1 across different batch sizes. In addition, when the batch size increases, the distance between two peaks of the distribution also increases, suggesting the enhanced statistical strength of PPSM gained by the combination of CVAE with the polarization regularization.

## 5. Conclusions

In this article, we propose a novel differentiable channel pruning method called polarization of probabilistic soft mask (PPSM). To capture the statistical behavior of the channel importance that is modeled in dynamic pruning under an input-aware manner, PPSM exploits variational inference to learn the posterior distributions of the masks and simultaneously classifies the filters into two clearly separated parts by leveraging a new polarization regularization, and thus, the channels with masks close to zero can be safely removed with little effect on network accuracy. We evaluated the performance of PPSM on several popular network architectures using CIFAR-10 and ImageNet datasets, and the results demonstrate our method performs competitive to the state of the arts. One of the limitations of PPSM lies in its low efficiency in learning the soft masks via the CVAE framework, and we plan to improve this in near future.

## Figures and Tables

**Table 1:** Setting of hyperparameters *λ* and *t* for ResNet56 on CIFAR-10 and ResNet50 on ImageNet.

Datasets	Network	*λ*	*t*	FLOPs ↓ (%)
CIFAR-10	ResNet56	0.0004	0.2	54.60
			0.8	66.84
			1.2	75.62

ImageNet	ResNet50	0.00005	−0.2	53.07
			0.2	65.91
			0.5	72.43

**Table 2:** Comparison results on the CIFAR10 dataset with VGG16, ResNet32, ResNet56, and ResNet110. Acc ↓ is the accuracy drop of the pruned model compared to the baseline model. FLOPs ↓ represent the pruning rate of FLOPs.

Network	Method	Baseline acc (%)	Pruned	Acc ↓ (%)	FLOPs ↓ (%)
VGG-16	HRank [11]	93.96	92.34	1.62	65.30
	SCP [14]	93.85	93.79	0.06	**66.23**
	**PPSM (ours)**	93.72	93.78	**−0.06**	66.20

ResNet32	LFPC [46]	92.63	92.12	0.51	52.60
	Wang et al. [47]	93.18	93.27	−0.09	49.00
	**PPSM (ours)**	93.19	93.31	**−0.12**	53.27
	LRF [27]	92.49	92.54	−0.05	62.00
	MainDP [15]	92.66	92.15	0.51	63.20
	**PPSM (ours)**	93.19	93.28	**−0.09**	**64.35**

ResNet56	Zhuang et al. [28]	93.80	93.83	−0.03	47.00
	HRank [11]	93.26	93.17	0.09	50.00
	LFPC [46]	93.59	93.34	0.25	52.90
	DMC [13]	93.62	93.69	−0.07	50.00
	SRR-GR [48]	93.38	93.75	**−0.37**	53.80
	SCP [14]	93.69	93.23	0.46	51.50
	DPFPS [49]	93.81	93.20	0.61	52.86
	Wang et al. [47]	93.69	93.76	−0.07	50.00
	**PPSM (ours)**	93.44	93.57	−0.13	**54.60**
	HRank [11]	93.26	90.72	2.54	74.10
	LRF-60 [27]	93.45	93.19	0.26	73.90
	**PPSM (ours)**	93.44	93.22	**0.22**	**75.62**

ResNet110	HRank [11]	93.50	92.65	0.85	68.60
	LFPC [46]	93.68	93.79	−0.11	60.30
	LRF [27]	93.76	94.34	**−0.58**	62.60
	**PPSM (ours)**	93.60	93.83	-0.23	**68.70**

The bold values are given to highlight the best-performing method in each performance metric.

**Table 3:** Comparison results of ResNet50 and MobileNet v2 on ImageNet. Pruned top-1 and pruned top-5 denote the top-1 and top-5 accuracy after the pruning. Top-1 ↓ and top-5 ↓ denote the accuracy drop of the pruned model when compared to the baseline model.

Network	Method	Pruned top-1(%)	Top-1 ↓ (%)	Pruned top-5(%)	Top-5 ↓ (%)	FLOPs ↓ (%)
ResNet50	GAL [16]	71.80	4.35	90.82	2.05	55.01
	Zhuang et al. [28]	75.63	0.52	—	—	54.00
	DMCP [18]	76.20	0.40	—	—	46.34
	DMC [13]	75.35	0.80	92.49	0.38	55.00
	HRank [11]	74.98	1.17	92.33	0.54	43.77
	GBN [17]	75.18	0.67	92.41	0.25	55.06
	SRR-GR [48]	75.11	1.02	92.35	0.51	55.10
	SCP [14]	75.27	0.62	92.30	0.68	54.30
	SCOP [50]	75.26	0.89	92.53	0.34	54.60
	LRF [27]	75.71	0.50	92.80	**0.02**	**56.40**
	DPFPS [49]	75.55	0.60	92.54	0.33	46.20
	**PPSM (ours)**	75.78	**0.35**	92.83	0.03	53.07
	GAL [16]	69.88	6.27	89.75	3.12	61.37
	HRank [11]	71.98	4.17	91.01	1.86	62.10
	CHIP [51]	75.26	0.89	92.53	0.34	62.80
	**PPSM (ours)**	75.43	**0.70**	**92.54**	**0.32**	**65.91**
	GAL [16]	69.31	6.84	89.12	3.75	72.86
	HRank [11]	69.10	7.05	89.58	3.29	**76.04**
	DMCP [18]	74.40	2.20	—	—	73.17
	CHIP [51]	73.30	2.85	91.48	1.39	76.70
	**PPSM (ours)**	74.59	**1.54**	92.30	**0.56**	72.43

MobileNet v2	AMC [7]	70.80	1.00	—	—	27.00
	Metapruning [52]	71.20	0.80	—	—	27.00
	DPFPS [49]	71.10	0.90	—	—	24.89
	**PPSM (ours)**	71.43	**0.45**	89.92	0.37	**28.69**

The bold values are given to highlight the best-performing method in each performance metric.

**Table 4:** Comparison between probabilistic and deterministic mask on the CIFAR-10 dataset. Acc ↓ is the accuracy drop of pruned model compared to the baseline model. FLOPs ↓ represent the pruning rate of FLOPs.

Network	Mask	Baceline acc (%)	Pruned acc (%)	Acc ↓ (%)	FLOPs ↓ (%)
ResNet32	Probabilistic	93.19	93.31	−0.12	53.27
			93.11	0.08	68.60
			93.00	0.19	74.40
	Deterministic	93.19	93.12	0.07	53.35
			92.93	0.26	69.59
			92.81	0.38	72.70

ResNet56	Probabilistic	93.44	93.57	−0.13	54.60
			93.35	0.09	66.84
			93.22	0.22	75.62
	Deterministic	93.44	93.24	0.20	54.30
			93.07	0.37	69.59
			92.91	0.53	75.16

## Data Availability

We evaluated the proposed method on two datasets: CIFAR-10 [40] and ImageNet ILSVRC 2012 [41].
